# Gastric outlet obstruction in an 11-year-old girl due to a pyloric diaphragm – A case report and a systematic literature review

**DOI:** 10.1016/j.ijscr.2025.110882

**Published:** 2025-01-14

**Authors:** Omran Janoud, Talal Abou Moughdib, Majd Hamed Nasser, Obaida Abo Fakher, Rani Saab, Houssain AL-Halabi

**Affiliations:** Faculty of Medicine, Damascus University, Damascus, Syria

**Keywords:** Gastric outlet obstruction, Pyloric diaphragm, General surgery

## Abstract

**Introduction:**

Congenital pyloric web or diaphragm are rare causes of Gastric Outlet Obstruction (GOO) after infancy, representing approximately 1 % of gastrointestinal obstructions. While it typically presents in the neonatal period with nonbilious vomiting, delayed diagnosis beyond infancy is exceptionally rare.

**Presentation of the case:**

An 11-year-old girl with presented with one-month history of postprandial vomiting. Her medical history was unremarkable and clinical examination and laboratory investigations were normal. A barium meal X-ray revealed narrowing at the gastric outlet. Endoscopy showed severe pyloric narrowing with undigested food in the stomach. Due to the severity of the stenosis, the endoscope could not traverse and surgical intervention was required.

**Discussion:**

Pyloric webs and diaphragms are considered rare causes of congenital GOO. Confirmation of the diagnosis requires an upper contrast study or endoscopy. Gastric outlet obstruction can manifest with various symptoms. The clinical onset varies depending on the underlying cause. We conducted a systematic literature review of all case reports and series focused on GOO patients over 10 years old due to webs or diaphragms. The review reveals that symptoms are varied and may persist for years before diagnosis due to the rarity of these anomalies and their nonspecific presentations. This review highlights the importance of follow-up period after treatment due to the risk of recurrence.

**Conclusion:**

This case emphasizes the importance of conducting endoscopy or abdominal X-rays with barium meals in evaluating persistent gastrointestinal symptoms, particularly in female patients.

## Highlights

• Congenital gastric outlet obstruction due to a web or diaphragm is very rare.
• Gastric outlet obstruction can manifest with various gastrointestinal symptoms.
• Symptoms may persist for years before a definitive diagnosis is made.
• Symptoms tend to manifest later in women compared to men as per our review.
• The paper introduces an unusual presentation of a pyloric diaphragm in an 11-year-old girl.

## 1 Introduction

Gastric outlet obstruction (GOO) in children is a clinical syndrome containing a spectrum of disorders, including mechanical obstructions and motility disorders. Mechanical obstructions, whether extrinsic or intrinsic, may be located in the distal stomach (prepyloric region), pyloric canal, or duodenum, especially in its first part. GOO is a common disorder and affects 2 to 5 per 1000 infants and children annually [1,2]. GOO can be classified in 3 groups:

1. Congenital intrinsic and extrinsic obstruction [Aplasia, Atresia, Diaphragm and web, Annular pancreas]
2. a) IHPS (congenital or infantile hypertrophic pyloric stenosis) b) Late-onset hypertrophic pyloric stenosis
3. Acquired causes (peptic ulcer, Chemical injury, Infectious causes, tumor and eosinophilic gastroenteritis) [2].

Congenital gastric outlet obstruction due to a web or diaphragm is a rare cause of GOO and an uncommon finding during endoscopy, it accounts for only about 1 % of all gastrointestinal obstructions [3]. This structural abnormality leads to a varying range of clinical presentations, based on the degree of obstruction. The diaphragm is a common form of type-1 atresia and can occur in the duodenum, antrum (most of the time), or pylorus, the pyloric location is the rarest [4,5]. Pyloric web, the most common type of pyloric atresia (PA), is generally detected during the neonatal period with nonbilious vomiting. Its delayed diagnosis is very rare [5]. Therefore, only a few cases report on pyloric diaphragm as a cause of gastric outlet obstruction in childhood [1]. In this study, we present a case of a 11-years old-girl who presented with severe pyloric stenosis and a delayed diagnosis of a pyloric diaphragm. The rarity of this situation, coupled with the systematic literature review conducted, renders this report unique and underscores the significance of considering gastric outlet obstruction as a potential cause of persistent gastrointestinal symptoms across all age groups.

This work was reported following the 2023 SCARE criteria [6].

## 2 Presentation of the case

A 11-year-old girl was admitted to our hospital with a one-month history of postprandial vomiting. The patient's past medical history was unremarkable, and aside from the current complaint, she did not have any significant medical conditions. The history revealed no specific behaviors that could predispose the patient to her current symptoms (e.g., abnormal eating behaviors). According to the patient's report, there has been a slight decrease in her condition over the previous weeks. The parents were divorced, and the staff addressed this situation to alleviate any psychological distress. Upon admission, vital signs were within normal limits for her age, and laboratory tests did not indicate any electrolyte disturbances. Complete blood count (CBC), liver function tests, and renal function tests were all within normal ranges. The patient was referred for medical management until a surgical consultation could be performed.

**Fig. 1:**
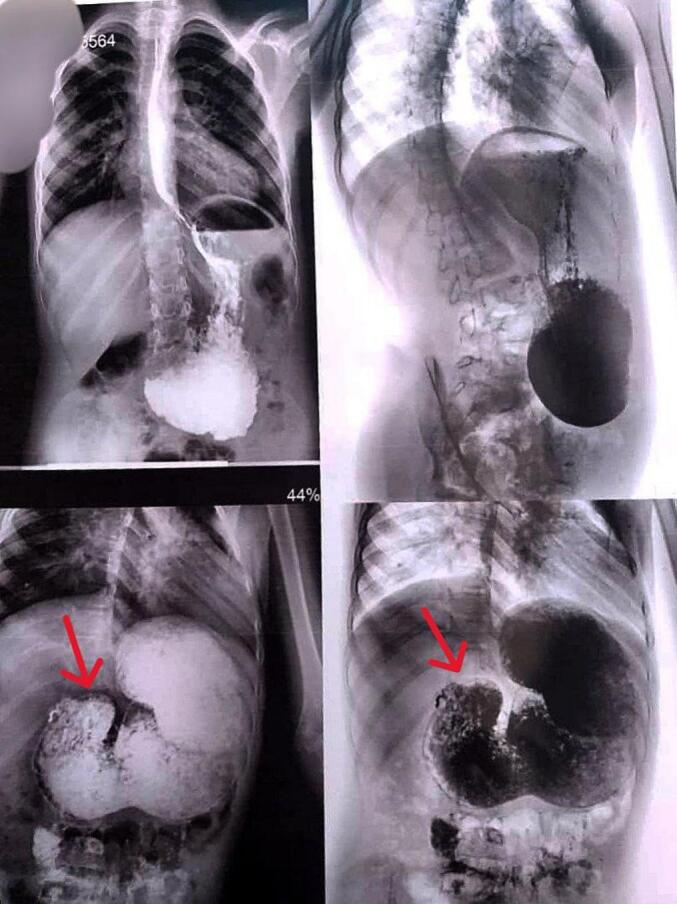
Barium upper gastrointestinal series illustrating the pyloric diaphragm with a stenotic opening (Indicated by the arrows).

Barium meal X-ray imaging revealed a narrowing at the level of the gastric outlet (Fig. 1). Differential diagnoses included gastric outlet obstruction due to foreign body, bezoar, antral web, or diaphragm. Endoscopy showed a normal esophagus and a stomach filled with undigested food, along with severe narrowing at the pylorus; however, the endoscope could not pass through this constriction. No ulcers were observed. Due to the severe stenosis, removing the diaphragm during endoscopy was not possible. As a result, a decision was made to proceed with surgical exploration.

**Fig. 2:**
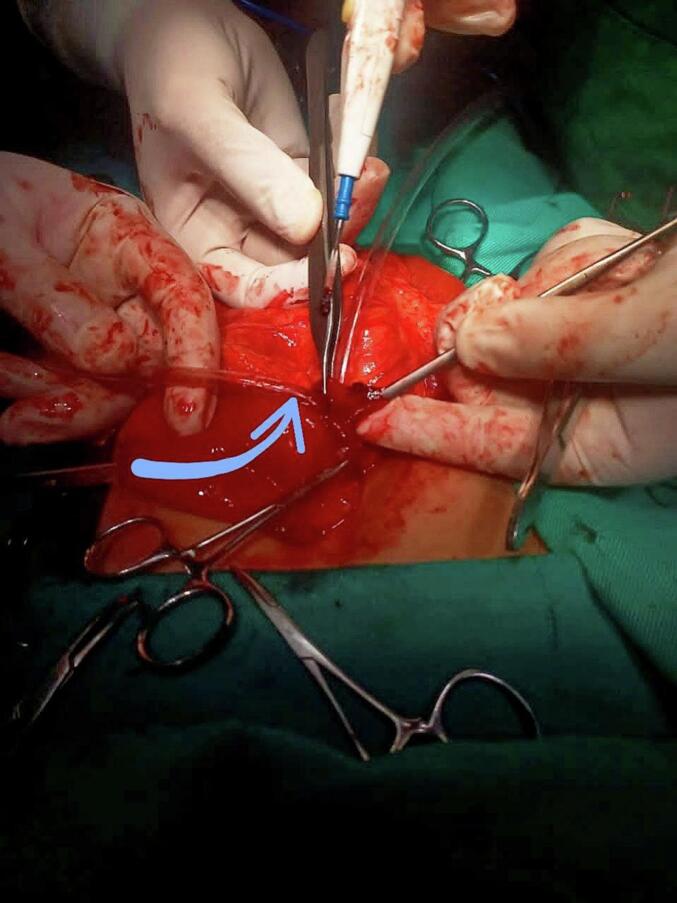
Intraoperative image depicting the insertion of a tube to assess the degree of stenosis; the tube (indicated by the arrow) was unable to traverse the stenosis.

**Fig. 3:**
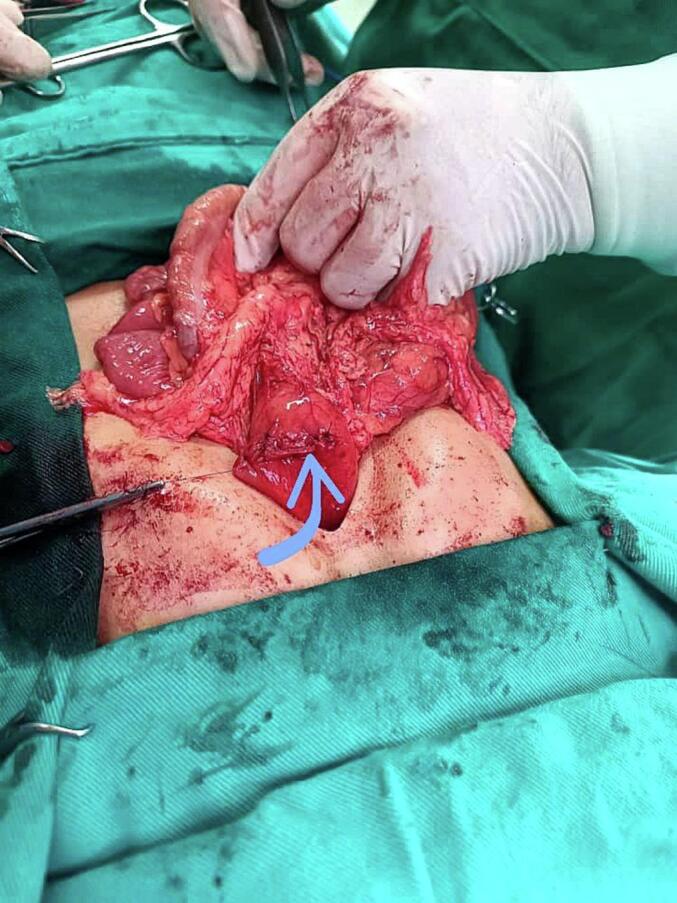
Image of the stomach demonstrating the closure of the longitudinal gastrotomy utilizing Heineke-Mikulicz pyloroplasty (indicated by the arrow).

An upper midline incision was performed to expose the stomach. A 2 cm incision on the greater curvature of the stomach allowed for exploration of the pylorus by inserting a tube through this incision (Fig. 2), which revealed severe narrowing at the pylorus and confirmed the presence of a diaphragm. The diaphragm was excised, and a longitudinal gastrotomy through the stenotic area was performed, followed by closure using Heineke-Mikulicz pyloroplasty techniques (Fig. 3). The surgical procedure was performed without complications, and no difficulties were encountered during its execution.

The patient was monitored in the hospital and discharged on postoperative day seven. After two weeks, she reported no symptoms. The patient gained nearly 2 kg during the 2 weeks after the operation.

## 3 Discussion

Pyloric webs and diaphragms are considered rare causes of congenital gastric outlet obstruction, and they are infrequently observed during endoscopy. These etiologies account for approximately 1 % of all gastrointestinal obstruction cases [7].

X-ray imaging may suggest such etiologies by revealing a large single gastric air bubble alongside a gasless, non-distended abdomen; however, gas may be visualized distal to a perforated web if present. Confirmation of the diagnosis typically necessitates an upper contrast study or endoscopy [8,9]. In our case, X-ray imaging revealed stenosis at the pylorus, prompting differential diagnoses that included foreign body ingestion, bezoar formation, pyloric web, or diaphragm. Endoscopy ruled out the presence of a foreign body, while surgical exploration confirmed the diagnosis of a pyloric diaphragm.

Although delays in diagnosis can occur, radiological, ultrasonographic, and endoscopic investigations are instrumental in facilitating timely identification [3].

Gastric outlet obstruction can manifest with various symptoms including nausea, vomiting, epigastric pain, early satiety, abdominal distention, abdominal mass formation, visible peristalsis, weight loss, and electrolyte imbalances. The most prevalent symptoms include epigastric pain, nausea and vomiting, abdominal distention, early satiety, and weight loss [10].

The onset of symptoms varies depending on the underlying cause; for instance, conditions such as gastric volvulus or corrosive injuries typically present rapidly compared to other etiologies that may exhibit a more insidious course [10]. This raises questions regarding the extent to which pyloric and antral webs and diaphragms may delay symptom presentation and when these etiologies should be considered in patients presenting with gastrointestinal symptoms.

A review of the literature reveals numerous cases documenting delays in diagnosing pyloric and antral webs or diaphragms as causes of vomiting and abdominal pain despite prolonged symptom duration prior to diagnosis. To investigate this delay further, we conducted a systematic literature review encompassing all case reports and series available on PubMed that reported gastric outlet obstruction due to webs or diaphragms in patients over 10 years old. We used the following search strategy:

((Antr*) OR (pylor*) OR (Pyloric Sphincter) OR (Pyloric Sphincters) OR (Sphincter, Pyloric) OR (Sphincters, Pyloric)) AND ((diaphragm) OR (web)) AND ((“Case Reports” [Publication Type]) OR (Case) OR (report) OR (Case Series) OR (Case Histories) OR (Case Study) OR (Case Studies))

We identified 455 publications but excluded 328 (37 were not in English; 18 were inaccessible; others were excluded based on publication type or age criteria). Ultimately, 27 studies were included in our review.

A summary of the findings can be found in Table 1. We excluded cases presented in the first table in the reference [7] due to the fact that the period of symptoms before diagnosis is not mentioned for these cases; subsequently, they are not relevant to our review. A review of significant findings from studies [7,[11], [12], [13], [14], [15], [16], [17], [18], [19], [20], [21], [22], [23], [24], [25], [26], [27], [28], [29], [30], [31], [32], [33], [34], [35], [36]] indicates that the mean age of patients was 49.69 years—suggesting these conditions can be diagnosed later in life rather than exclusively during infancy. In the majority of cases reviewed, gastroscopy was established as the gold standard for diagnosis.

**Table 1:** A review of all included cases published on Pubmed using the mentioned search strategy.

Study ID	The number of the case (for case series)	Sex	Age (years)	Presenting symptoms	Period of symptoms before diagnosis	The Way the Final Diagnosis was established	The treatment	Follow-up
Guo (2024)		M	11	Intermittent vomiting	8-year	Gastroscopy	Endoscopic treatment	2 years
Gehwolf (2019)	4	M	11	Nutritional deficiencies & Avoidance of solid food	Since early childhood	Not clearly defined	Endoscopic treatment	1.5 years
Guo (2019)		M	33	Postprandial epigastric distress & repeated nonbilious vomiting	10 years	Barium swallow	Endoscopic treatment (3times)	3 months
Akbaş (2019)		M	25	N/A	Not mentioned	Gastroscopy	Not Mentioned	Not Mentioned
Morales (2017)	1	M	60	Gastroesophageal reflux, Odynophagia and Retrosternal pressure after eating solid foods	3 months	Gastroscopy	Endoscopic treatment	5 years
	2	F	80	Dysphagia to solids and liquids, persistent nausea and vomiting, and abdominal fullness	Not mentioned	Gastroscopy	Endoscopic treatment	Not Mentioned
	3	M	68	Nausea, frequent regurgitation and early satiety with a 90-pound weight loss	10 years	Gastroscopy	Endoscopic treatment	3 weeks
	5	F	81	Persistent nausea and vomiting	Not mentioned	Gastroscopy	Endoscopic treatment (2 times)	8 weeks between the 2 endoscopic interventions. No follow up period mentioned after the 2nd procedure
Salah (2013)		M	11	Nausea, abdominal pain, and failure to thrive	Not mentioned	Gastroscopy	Endoscopic treatment	9 months
Gul (2011)		M	103	Hematemesis, nausea, vomiting, and decreased oral intake	a few months	Gastroscopy	Endoscopic treatment	Not Mentioned
Lui (2000)		F	10	Intermittent abdominal pain and vomiting	6 months	Gastroscopy	Endoscopic treatment	Not Mentioned
Maldonado (1998)		F	40	GERD, upper quadrant abdominal pain, and occasional post-prandial vomiting	1 year	Gastroscopy (other investigations were: Endoscopic ultrasonography, gastric emptying study)	Endoscopic treatment	5 months
Sikorski (1994)	1	M	27	Dyspepsia	Not mentioned	Gastroscopy	Not Mentioned	Not Mentioned
	2	F	70	Postprandial epigastric pain	Not mentioned	Gastroscopy	Not Mentioned	Not Mentioned
Travis (1990)		F	69	Dyspepsia	3 months	Gastroscopy	Endoscopic treatment	Not Mentioned
Al-Kawas (1988)		F	64	Postprandial midabdominal pain associated with foul smelling belching and followed by watery diarrhea.	4 years	Gastroscopy	Endoscopic treatment (with YAG laser)	6 weeks period (relapse)
Blazek (1987)	6	M	11	Cyclical vomiting, lose weight, epigastric pain 2 to 3 h after meals lasting 30 min.	1 year	Gastroscopy	Was not clearly defined	3 days
Berr (1985)		F	14	Epigastric discomfort, prandial fullness, cramping epigastric pain, and occasional exacerbations with vomiting of gastric contents and watery diarrhea (5 stools/day).	1 year	Gastroscopy	Endoscopic treatment	9 months
Pederson (1984)		M	65	Nausea and vomiting	Not mentioned	Barium swallow	Open surgery	Not Mentioned
Huggins (1982)		F	61	Postprandial fullness with occasional nausea and vomiting.	2 months	Gastroscopy	Conservative therapy	Not Mentioned
Mitchell (1979)	1	F	70	Anorexia, occasional vomiting and weight loss	9 months	Gastroscopy	Open surgery	6 months
	2	F	77	General malaise and vomiting after meals	Not mentioned	Gastroscopy	Open surgery	6 months
	3	F	71	Epigastric discomfort. During the year before presentation, the pain became more severe, particularly following meals. The patient was nauseous but had never vomited.	40 years	Gastroscopy	Open surgery	5 months
Javier-Gabriel (1977)		F	41	Intermittent abdominal pains	since childhood	Gastroscopy	Open surgery	Not Mentioned
Cho (1976)	1	F	50	Indigestion and epigastric fullness after meals	5 years	Gastroscopy		
	2	M	57	Asymptomatic (the pateint was being followed fpr hypertension)	Not mentioned	Gastroscopy		
	3	F	55	Recent onset of a seizure disorder and hematemesis	Not mentioned	Gastroscopy	Open surgery	Not Mentioned
Rona (1975)				The gastric outlet obstruction in this case did not produce symptoms until a lifespan of more than eight decades				
Hait (1972)		F	67	Epigastric fullness, frequently associated with nausea and vomiting, but only rarely with pain lost 2.7 kg (6 lb.)	4 months	Gastroscopy	Open surgery	Not Mentioned
Robinson (1967)		M	65	Intermittent indigestion and occasional vomiting	7 years	Barium swallow	Open surgery	1 year
Diethrich (1965)		F	64	Epigastric discomfort, numerous episodes of midepigastric fullness and right upper quadrant aching since childhood, usually occurring about 1 h after eating. During the twelve months prior to her hospitalization, she had the additional complaint of vomiting after the intake of food.	Lifelong	Gastroscopy	Open surgery	Not Mentioned
YOUNG (1961)		F	53	Comatose patient	several years	Surgical exploration	Open surgery	2 year
CHAMBERLAIN (1959)	1	F	55	Fullness after meals, lost just over 21 St. (15.9 kg.) in weight. Her appetite was variable, and sometimes she had eaten reasonably well.	8 months	Surgical exploration	Open surgery	Not Mentioned
	2	M	52	Intermittent ache in the epigastrium, which had become worse during the previous six months. he had vomited two to three times a week, mainly at night. He had lost 14 lb. (6.4 kg.) in weight during the previous six months, and his appetite was poor.	5 years	Surgical exploration	Open surgery	Not Mentioned
GROSS (1952)		F	35	Intermittent vomiting	several years	Surgical exploration	Open surgery	Not Mentioned
SAMES (1949)		F	40	Epigastric pain associated with the taking of food	6 years	Barium swallow	Open surgery	Not Mentioned

M: male, F: female.

The analysis of various studies indicates that many patients experience prolonged symptoms, often exceeding a year, before receiving a diagnosis of pyloric or antral webs and diaphragms [[11], [12], [13], [14],^20^,^23^,^24^,[27], [28], [29],[31], [32], [33], [34], [35], [36], [37]]. Moreover, an additional case [38] (abstract only) indicated that the patient did not experience symptoms until after a lifespan exceeding eight decades. In all these cases, no clear cause for the formation of the diaphragm or web was identified. This point is significant as it underscores the extent to which diagnosis may be delayed despite the presence of symptoms for many years. In case [12], the patient received conservative treatment at a local hospital two or three times annually before a diagnosis was established and endoscopic intervention was performed.

In case [18], the diaphragm developed without producing symptoms, leading to no intervention as per the authors' report. Furthermore, the patient described in reference [26] underwent conservative therapy; however, many details regarding follow-up and response were not provided. Other cases were treated surgically or during endoscopy. Among patients who were followed up, a substantial proportion remained asymptomatic. However, the patient reported in reference [14] along with the first, second, and fifth cases in reference [37] as well as the patient in reference [11] experienced relapse after varying periods of endoscopic treatment. Conversely, the third patient reported in reference [27] died during follow-up; a metastatic adenocarcinoma was discovered during surgery, which was determined to be the cause of death. These findings emphasize the importance of documenting a follow-up period post-treatment due to potential recurrence.

Additionally, nearly two-thirds of patients were female (n1 = 21), while males constituted approximately one-third (n2 = 13). Consequently, it appears that symptoms associated with pyloric and antral webs and diaphragms tend to manifest later in women compared to men. Symptoms experienced by patients prior to diagnosis included postprandial vomiting (18 patients), postprandial fullness (7 patients), postprandial discomfort or abdominal pain (13 patients), along with other symptoms such as nutritional deficiencies and avoidance of solid food, dysphagia, dyspepsia, hematemesis, gastroesophageal reflux, weight loss (5 patients), and poor appetite.

## 4 Conclusion

In conclusion, manifestations of pyloric and antral webs and diaphragms in individuals over ten years can vary significantly among individuals; however, the most common manifestations include nausea, vomiting, and intermittent abdominal pain. Numerous cases documented in the literature indicate that symptoms may persist for years before a definitive diagnosis is made. This highlights the necessity of conducting endoscopy or abdominal X-rays with barium meals to investigate these etiologies as contributing factors, particularly in female patients presenting with gastrointestinal symptoms.

## Ethics statement

Ethical approval from our hospital is not required when publishing a single case report.

## Consent for publication

Written informed consent was obtained from the patient's parents/legal guardian for publication and any accompanying images. A copy of the written consent is available for review by the Editor-in-Chief of this journal on request.

## Funding

N/A.

## Author contribution

All authors have read and approved the manuscript. O.J. performed the systematic literature review, extracted the data and analyzed it. Writing - original draft: (T.A., O.J. & M.H.N.) Writing - review & editing (O.J., T.A., M.H.N., O.A., R.S., & H.A.), Data Curation (M.H.N.). All authors contributed substantially to the work and revised the manuscript critically for important intellectual content. All authors gave final approval of the version submitted for publication.

## Research registration

The research does not need any registration.

## Declaration of competing interest

The authors declare that they have no competing interests.

## Data Availability

N/A.
